# New Technologies for IBD Endoscopy

**DOI:** 10.3390/jcm15072539

**Published:** 2026-03-26

**Authors:** Cristina Bezzio, Valeria Farinola, Giuseppe Privitera, Arianna Dal Buono, Roberto Gabbiadini, Laura Loy, Gianluca Franchellucci, Erica Bartolotta, Giulia Migliorisi, Alessandro Armuzzi

**Affiliations:** 1 Gastroenterology and Digestive Endoscopy Unit, IRCCS Humanitas Research Hospital Department of Gastroenterology, Rozzano, 20089 Milan, Italy; valeria.farinola@humanitas.it (V.F.); giuseppe.privitera@humanitas.it (G.P.); arianna.dalbuono@humanitas.it (A.D.B.); erica.bartolotta@humanitas.it (E.B.); giulia.migliorisi@humanitas.it (G.M.); alessandro.armuzzi@hunimed.eu (A.A.); 2 Department of Biomedical Sciences, Humanitas University, 20072 Milan, Italy; roberto.gabbiadini@humanitas.it (R.G.); laura.loy@humanitas.it (L.L.); gianluca.franchellucci@humanitas.it (G.F.); 3 Department of Internal Medicine and Medical Therapeutics, University of Pavia, 27100 Pavia, Italy

**Keywords:** inflammatory bowel disease, ulcerative colitis, Crohn’s disease, endoscopy, image-enhanced endoscopy, confocal laser endomicroscopy, artificial intelligence, dysplasia surveillance, molecular imaging

## Abstract

**Background:** Endoscopic assessment is central to the management of inflammatory bowel disease (IBD), particularly within treat-to-target strategies. However, conventional high-definition white-light endoscopy (HD-WLE) is limited by interobserver variability and its inability to reliably reflect microscopic inflammation or predict long-term outcomes. Over the last decade, multiple technological innovations have reshaped the role of endoscopy in both disease activity monitoring and dysplasia surveillance. **Methods:** This narrative review provides a comprehensive and clinically oriented overview of emerging endoscopic technologies in IBD, including image-enhanced endoscopy, ultra-high-magnification techniques, artificial intelligence (AI), and molecular imaging. We discuss their diagnostic performance, prognostic implications, and potential integration into clinical practice. **Results:** Image-enhanced endoscopy improves visualization of subtle mucosal and vascular alterations and demonstrates stronger correlation with histological activity compared with HD-WLE alone. Confocal laser endomicroscopy and endocytoscopy enable in vivo microscopic assessment of epithelial architecture and barrier integrity, redefining remission beyond macroscopic healing. AI systems have shown expert-level performance in grading inflammatory severity in ulcerative colitis and high sensitivity in capsule endoscopy for Crohn’s disease, supporting objective and reproducible assessment. In surveillance, targeted high-definition inspection has replaced random biopsies, while adjunctive optical and AI-based tools enhance lesion detection and characterization. Molecular imaging introduces a predictive dimension by enabling visualization of drug–target engagement and dysplasia-specific pathways. **Conclusions:** Endoscopy in IBD is evolving from a descriptive modality toward a multimodal precision tool integrating enhanced imaging, AI-driven standardization, and molecular profiling. Although further validation and cost-effectiveness studies are required, these innovations have the potential to improve therapeutic stratification, surveillance strategies, and long-term patient outcomes.

## 1. Introduction

Inflammatory bowel diseases (IBD), encompassing ulcerative colitis (UC) and Crohn’s disease (CD), are chronic, immune-mediated inflammatory disorders of the gastrointestinal tract characterized by a relapsing–remitting course and a substantial impact on patients’ quality of life [1]. Over the past decade, their clinical management has undergone a profound transformation, shifting from a symptom-driven approach to a structured treat-to-target (T2T) strategy, as formalized by the STRIDE-II consensus [2]. This paradigm emphasizes the achievement of objective therapeutic endpoints aimed at modifying the natural history of disease. While mucosal healing (MH) remains the established therapeutic goal, increasing evidence supports deeper targets, including histological healing (HH) in UC and transmural healing (TH) in CD, as more accurate predictors of sustained steroid-free remission, reduced relapse rates, and decreased hospitalization [3,4,5,6].

Within this framework, endoscopy plays a central role in diagnosis, disease stratification, monitoring of therapeutic response, and surveillance for neoplasia [7]. High-definition white-light endoscopy (HD-WLE) has significantly improved image resolution compared to standard-definition systems and remains the cornerstone of routine practice [8]. However, despite these technological advances, conventional endoscopy is increasingly recognized as insufficient to meet the demands of precision medicine.

First, the assessment of endoscopic disease activity remains inherently subjective. Widely adopted scoring systems—such as the Mayo endoscopic score (MES) and the ulcerative colitis endoscopic index of severity (UCEIS) in UC, and the Crohn’s disease endoscopic index of severity (CDEIS) and the simple endoscopic score for Crohn’s disease (SES-CD) in CD—are limited by inter- and intra-observer variability, particularly in cases of mild-to-moderate disease activity [9,10,11,12,13,14,15,16,17,18,19]. Even among experienced endoscopists, reproducibility remains suboptimal, underscoring the difficulty of achieving standardized and objective quantification of inflammation based solely on human interpretation [20].

Second, a well-documented discordance exists between endoscopic remission and histological activity. Endoscopic healing does not necessarily equate to microscopic remission; approximately one-third of UC patients in apparent endoscopic remission exhibit persistent histological inflammation, which is strongly associated with subsequent clinical relapse [21,22]. Similarly, in ileal CD, histological activity may persist despite mucosal healing on endoscopy, with significant implications for long-term outcomes [23,24]. These findings highlight a critical “microscopic gap,” whereby HD-WLE underestimates the true burden of disease and fails to reliably predict histological healing.

Third, conventional ileocolonoscopy leaves a diagnostic blind spot in the proximal small bowel, particularly relevant in CD, where disease distribution is often patchy and may involve segments beyond the reach of standard endoscopy. Proximal small bowel involvement has been identified as an independent predictor of a disabling disease course and increased relapse risk [25]. Although cross-sectional imaging modalities such as magnetic resonance enterography (MRE) and intestinal ultrasound (IUS) provide valuable information on transmural and extraluminal disease [26], they may miss subtle superficial mucosal lesions. Small bowel capsule endoscopy (SBCE), supported by validated scoring systems such as the Lewis score (LS) and the capsule endoscopy Crohn’s disease activity index (CECDAI), offers a more sensitive evaluation of mucosal inflammation and is increasingly integrated into treat-to-target strategies [8,27,28,29,30].

Collectively, these limitations—operator-dependent variability, microscopic–macroscopic discordance, and incomplete assessment of disease extent—have driven a technological renaissance in IBD endoscopy. Advanced image-enhanced endoscopy (IEE), including dye-based chromoendoscopy and virtual chromoendoscopy, aims to improve visualization of subtle mucosal and vascular patterns and to approximate histological assessment [31,32,33,34,35,36,37,38,39,40,41,42,43,44,45,46,47,48,49]. Ultra-high-magnification techniques such as confocal laser endomicroscopy (CLE) and endocytoscopy (EC) enable in vivo microscopic evaluation of epithelial architecture and barrier integrity, narrowing the gap between endoscopy and histopathology [50,51,52,53,54,55,56,57,58,59,60,61,62,63,64,65,66,67]. Simultaneously, artificial intelligence (AI) is emerging as a powerful tool to standardize scoring systems, reduce observer variability, and provide objective, reproducible metrics of disease activity across ileocolonoscopy and capsule endoscopy [68,69,70,71,72,73,74,75,76,77,78,79,80,81,82,83,84,85,86,87,88,89,90,91,92,93,94,95,96]. At the most advanced frontier, molecular endoscopy leverages fluorescent probes to visualize specific pathogenic targets, offering the potential to predict therapeutic response and personalize biologic treatment strategies [97,98,99,100,101,102,103,104,105,106,107].

**Figure 1:**
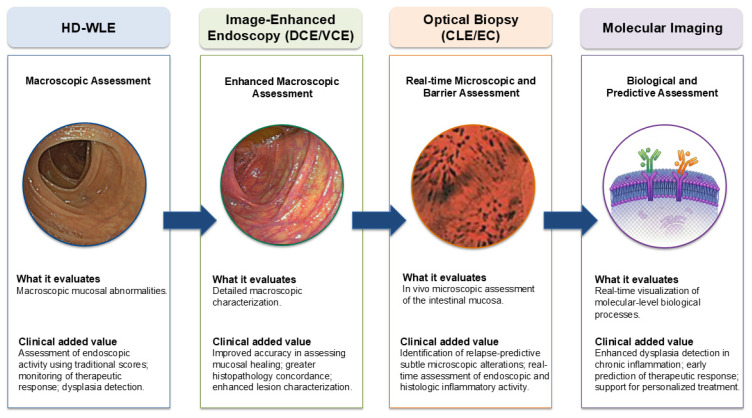
Evolution of endoscopic technologies in inflammatory bowel disease (IBD): from macroscopic to biologically driven assessment. **Abbreviations: CLE**, confocal laser endomicroscopy; **DCE**, dye-based chromoendoscopy; **EC**, endocytoscopy; **HD-WLE**, high-definition white-light endoscopy; **VCE**, virtual chromoendoscopy.

This review provides a comprehensive and critical update on these emerging technologies, examining their clinical applications, strengths, and current limitations in both disease activity monitoring and dysplasia surveillance. By integrating enhanced imaging, real-time microscopic assessment, AI-driven quantification, and molecular targeting, modern endoscopy is evolving from a descriptive diagnostic modality into a functional and predictive instrument capable of guiding precision medicine in IBD (Figure 1).

High-definition white-light endoscopy (HD-WLE) provides baseline macroscopic evaluation of mucosal appearance. Image-enhanced endoscopy (IEE), including dye-based chromoendoscopy (DCE) and virtual chromoendoscopy (VCE), improves contrast and visualization of mucosal and vascular patterns, refining detection of subtle inflammatory changes while remaining surface-based. Optical biopsy techniques—confocal laser endomicroscopy (CLE) and endocytoscopy (EC)—enable real-time microscopic assessment of epithelial architecture and barrier integrity, narrowing the endoscopic–histologic gap. Molecular imaging represents the highest level of diagnostic depth by visualizing specific molecular targets and drug–target engagement, introducing a predictive dimension to endoscopic assessment. Overall, these modalities represent complementary and progressively deeper layers of evaluation, moving from morphology to microstructure, function, and molecular profiling.

## 2. Disease Activity Assessment: Current Standards and Unmet Needs

### 2.1. High-Definition White-Light Endoscopy and Scoring Systems

HD-WLE remains the cornerstone for evaluating disease activity and therapeutic response in IBD [8]. Although it provides improved resolution compared with standard-definition systems, its diagnostic performance is constrained by subjectivity and limited correlation with microscopic inflammation.

In UC, the MES is widely used due to its simplicity but lacks formal validation and is prone to subjective interpretation, particularly in intermediate grades [12,13]. The UCEIS, based on vascular pattern, bleeding, and erosions/ulcers, offers improved reproducibility and stronger correlation with histological findings and clinical outcomes [14,15,16].

In CD, disease activity is assessed using the CDEIS and the SES-CD [17]. While CDEIS provides a detailed segmental assessment, its complexity limits routine use [18]. SES-CD simplifies evaluation into four domains, facilitating clinical applicability [19]. Despite these structured indices, interobserver variability remains substantial, particularly in mild-to-moderate disease, reflecting the intrinsic limits of visually based assessment [9,10,11,20].

### 2.2. The Microscopic and Small Bowel Gaps

Endoscopic remission does not necessarily correspond to histological healing. In UC, up to 30% of patients with endoscopic remission demonstrate persistent microscopic inflammation, which is associated with increased relapse risk [21,22]. Similar discordance has been reported in ileal CD, where histological activity may persist despite apparent mucosal healing [23,24]. These findings underscore the inability of HD-WLE to reliably predict deep remission.

In CD, conventional ileocolonoscopy also fails to assess the proximal small bowel, where involvement is more frequent than previously recognized and independently associated with a disabling disease course [25]. SBCE allows detection of subtle mucosal lesions that may be missed by cross-sectional imaging modalities such as MRE or IUS [26]. Validated indices, including the LS and the CECDAI, provide objective measures of inflammatory burden within a treat-to-target framework [27]. The development of pan-enteric capsule endoscopy (PCE), such as the PillCam Crohn’s system, enables near-complete visualization of the gastrointestinal tract in a single examination, improving disease mapping and monitoring [28,29]. However, capsule endoscopy remains limited by lower specificity, lack of histologic sampling, and the risk of retention in patients with strictures [30].

## 3. Enhanced Macroscopic Imaging: Image-Enhanced Endoscopy (IEE)

The limitations of conventional white-light endoscopy in accurately reflecting microscopic inflammation have driven the development of IEE, aimed at improving visualization of mucosal architecture and vascular patterns. IEE includes DCE and VCE, both designed to enhance contrast and better approximate histological activity [32,33].

### 3.1. Dye-Based Chromoendoscopy (DCE)

DCE relies on the topical application of chemical agents to enhance mucosal detail. Contrast agents such as indigo carmine accumulate within mucosal grooves and pits, highlighting surface irregularities without penetrating the epithelium. In contrast, absorptive dyes such as methylene blue or crystal violet are taken up by epithelial cells, improving visualization of microstructural alterations and allowing differentiation between inflamed and regenerative mucosa [32].

Compared with HD-WLE, DCE has demonstrated improved concordance with histopathological assessment of inflammation and more accurate delineation of disease extent [33]. In a prospective study, Ibarra-Palomino et al. showed that DCE with methylene blue or indigo carmine enhanced detection of subtle inflammatory changes in UC, increasing endoscopic–histologic agreement [34]. However, evidence supporting its role specifically for inflammatory burden assessment remains limited, as DCE has historically been used primarily for dysplasia surveillance [35].

Despite its diagnostic benefits, DCE is constrained by longer procedure times, uneven dye distribution, and the need for dedicated training, limiting its widespread adoption in routine monitoring [36].

### 3.2. Virtual Chromoendoscopy (VCE)

To overcome the logistical challenges of dye application, virtual chromoendoscopy technologies have been developed. These systems enhance mucosal and vascular contrast through optical filtering or digital post-processing, without the need for exogenous dyes [33,108].

Optical-based systems, such as narrow-band imaging (NBI; Olympus) and blue laser imaging (BLI; Fujifilm), utilize narrow wavelengths in the blue (415 nm) and green (540 nm) spectrum, corresponding to hemoglobin absorption peaks. This enhances visualization of superficial capillaries and mucosal vascular patterns, which are early indicators of inflammation or healing [37,38]. Beyond contrast enhancement, NBI has been shown to assess mucosal angiogenesis [39].

Assessment of mucosal vascular patterns (MVPs) using NBI has demonstrated superior predictive performance compared with white-light endoscopy (WLE) for histological healing (Nancy index ≤1) and for 12-month clinical relapse in UC, potentially reducing reliance on random biopsies [40]. Similarly, magnifying NBI features correlate significantly with histopathological findings in UC (*p* < 0.01), supporting their prognostic relevance [41].

Digital post-processing systems, including i-Scan (Pentax) and linked color imaging (LCI; Fujifilm), enhance specific color tones in real time while maintaining white-light illumination. In a randomized controlled trial involving 78 IBD patients, i-Scan demonstrated stronger concordance with histology compared with HD-WLE, particularly in defining disease extent (92.3% vs. 48.7%, *p* = 0.0009) and inflammatory activity (89.7% vs. 53.9%, *p* = 0.066) [42].

LCI enhances color contrast by intensifying red inflammatory areas and whitening healed mucosa. In UC patients in clinical remission, LCI identified minimal residual inflammation undetectable by HD-WLE and predicted disease relapse with high accuracy [43,44]. Uchiyama et al. further proposed an LCI-based classification (LCI-A/B/C) stratifying mucosal redness. This system demonstrated excellent interobserver agreement and reclassified a substantial proportion of MES 0–1 areas into higher-risk categories, suggesting improved discrimination within endoscopic remission [45]. Building on this concept, the SOUL study introduced the LCI Index, combining redness, inflamed area, and lymphoid follicles to predict histological healing in UC patients with MES ≤1. LCI-based scores effectively identified residual histological activity not captured by conventional WLE grading [46].

The detailed visualization afforded by VCE has also led to the development of dedicated scoring systems. The PICaSSO (Paddington International Virtual ChromoendoScopy ScOre) evaluates mucosal and vascular architecture, generating a composite score (0–15) [47]. Initially validated using i-Scan, PICaSSO has demonstrated strong correlation with established histologic indices, including the Robarts histopathology index (RHI) and the Nancy histological index (NHI), across multiple platforms (including NBI and LCI) [48]. Correlation coefficients exceeding 0.90 have been reported, surpassing traditional indices such as MES and UCEIS. Furthermore, PICaSSO predicts clinical outcomes at 6 and 12 months with performance comparable to histological assessment, supporting its utility in therapeutic decision-making [49].

### 3.3. Bridging the Macroscopic–Microscopic Gap

Overall, image-enhanced endoscopy improves visualization of subtle mucosal and vascular alterations and demonstrates stronger concordance with histology compared with HD-WLE alone. While DCE remains limited by practical constraints, VCE provides a more feasible and scalable approach, particularly in UC, where vascular pattern analysis and dedicated scoring systems such as PICaSSO and LCI-based indices help bridge the macroscopic–microscopic gap.

Although VCE significantly improves macroscopic evaluation, it remains limited to surface assessment, reinforcing the need for ultra-high-magnification techniques capable of real-time microscopic analysis.

## 4. Optical Biopsy and Barrier-Level Assessment

The progressive recognition of the microscopic gap between endoscopic and histological remission has stimulated the development of ultra-high-magnification techniques capable of in vivo histologic assessment. CLE and EC represent the most advanced modalities in this field, enabling real-time evaluation of epithelial architecture, inflammatory infiltrates, and barrier integrity [50].

### 4.1. Confocal Laser Endomicroscopy (CLE)

CLE provides real-time microscopic imaging of the intestinal mucosa using a low-power laser and a fluorescent contrast agent, typically intravenous fluorescein. Probe-based CLE (pCLE), the most widely adopted modality, is introduced through the working channel of a standard endoscope and allows targeted optical biopsies at magnifications up to 1000× [51].

Beyond structural assessment, pCLE enables functional evaluation of epithelial barrier integrity. Increased epithelial gap density and fluorescein leakage have been identified as early markers of barrier dysfunction. Turcotte et al. demonstrated that elevated epithelial gap density in the terminal ileum predicted hospitalization or surgery in IBD patients (log-rank *p* = 0.02), suggesting prognostic relevance beyond conventional endoscopic findings [52]. Similarly, the prospective ERIca trial showed that endomicroscopic evidence of barrier healing was superior to both endoscopic and histologic remission in predicting long-term favorable outcomes in UC and CD [53].

To standardize interpretation, several scoring systems have been proposed. The Watson score evaluates fluorescein leakage and micro-erosions as indicators of barrier dysfunction, showing high concordance with histology and strong predictive value for relapse (*p* < 0.001) [54,55]. In UC, the ENHANCE index was developed to assess histological activity in vivo; pCLE features such as crypt distortion, vessel dilation, and fluorescein leakage correlated with validated histologic indices, with an overall accuracy of approximately 80%. An ENHANCE score ≤1 demonstrated a high negative predictive value (93.1%) for excluding microscopic inflammation, functioning as a real-time surrogate of the Nancy index [56].

In CD, the Crohn’s disease endomicroscopic activity score (CDEAS) evaluates crypt architecture, microvascular alterations, and inflammatory features, achieving an accuracy of 87% in predicting histologic remission [57].

Despite these promising results, the implementation of CLE remains limited by equipment costs, the need for intravenous contrast, and the steep learning curve required for image interpretation [51].

### 4.2. Endocytoscopy (EC)

EC represents the highest level of optical magnification in gastrointestinal endoscopy (approximately 450×–1400×), allowing direct visualization of epithelial cells and inflammatory infiltrates following topical application of vital dyes such as methylene blue or crystal violet [50].

In UC, EC has shown strong correlation with conventional histology. Endocytoscopic narrow-band imaging (EC-NBI) demonstrated significant concordance with histologic inflammation (r = 0.87, *p* < 0.01) [59]. In patients with clinical and endoscopic remission (MES 0), EC predicted histologic remission with 77% sensitivity and 97% specificity, with substantial agreement with the Geboes score (κ = 0.72) [60].

Several scoring systems have been developed to standardize EC interpretation. The endocytoscopy system score (ECSS) assesses crypt morphology and microvascular visibility, demonstrating strong correlation with histology (ρ = 0.713) and high interobserver agreement (κ = 0.79) [61]. More recently, the ELECT (ErLangen Endocytoscopy in ColiTis) score showed strong correlation with both the Robarts histopathology index (r = 0.70) and the Nancy index (r = 0.73), with high sensitivity (88%), specificity (95.2%), and AUROC (0.916) for histologic activity [62]. In direct comparison studies, ELECT outperformed ECSS in identifying histological remission, supporting its robustness as a real-time assessment tool [63].

Beyond grading inflammation, EC has demonstrated prognostic value. Persistence of microvascular abnormalities or goblet cell depletion in patients with apparent endoscopic remission predicts higher relapse rates. Takishima et al. reported significantly higher cumulative relapse in MES 0 patients with goblet cell depletion compared with those with preserved goblet cells (19% vs. 5%, *p* = 0.02), highlighting the importance of barrier integrity assessment [64,65].

In CD, the discontinuous and transmural nature of inflammation limits standardization; however, EC has demonstrated high specificity (>90%) for identifying inflammatory cells in vivo and substantial interobserver agreement (κ = 0.61–0.78) [66].

### 4.3. Molecular Validation of Barrier Healing

The concept of barrier healing has recently received molecular validation. In the multicenter endo-histo-barrier-omics study, Iacucci et al. demonstrated that architectural abnormalities detected by ultra-high-magnification EC and pCLE correlate with molecular markers of barrier dysfunction. In UC, epithelial crypt distortion and goblet cell depletion moderately correlated with Claudin-2 overexpression, whereas in CD, vascular alterations correlated with PV-1 expression, a marker of vascular permeability. Importantly, integration of imaging features—standardized through AI—with molecular profiling significantly outperformed conventional endoscopy in predicting major adverse outcomes (hazard ratio 3.4 in UC; hazard ratio 2.9 in CD) [67].

Collectively, these data position optical biopsy technologies as tools not only for real-time histologic approximation but also for functional and prognostic assessment, potentially redefining remission from a purely macroscopic concept to a barrier-based endpoint.

However, the complexity of interpreting high-resolution imaging further underscores the potential role of artificial intelligence in standardizing real-time assessment.

## 5. Artificial Intelligence in IBD Endoscopy

The increasing need for objective, reproducible, and scalable endoscopic assessment has positioned AI as a transformative tool in IBD management. Traditional endoscopic scoring systems are inherently operator-dependent and affected by interobserver variability, potentially influencing therapeutic decisions and clinical trial outcomes [12]. AI enables a transition from qualitative visual interpretation to quantitative, standardized, and potentially automated evaluation [72].

Machine learning (ML) algorithms identify patterns within structured datasets to support prediction models, whereas deep learning (DL), particularly convolutional neural networks (CNNs), allows automated extraction of relevant spatial features directly from endoscopic images and videos [73,74]. In IBD, AI applications have focused on two main domains: disease activity assessment and dysplasia detection.

### 5.1. AI for Disease Activity Assessment

In UC, early CNN-based systems demonstrated performance comparable to expert central readers in grading endoscopic severity. Stidham et al. reported an AUROC of 0.966 for distinguishing remission from moderate-to-severe disease using the MES, with agreement comparable to expert reviewers (κ = 0.84) [75]. Takenaka et al. subsequently developed a deep neural network capable of predicting both endoscopic remission (UCEIS) and histological remission (Geboes score) from still images, achieving high accuracy (90.1% and 92.9%, respectively) [76].

The extension from still images to full-length video analysis further enhanced clinical applicability. Gottlieb et al. demonstrated excellent agreement between AI and human central readers in grading MES and UCEIS in randomized controlled trial videos (QWK 0.844–0.855) [77]. Fan et al. developed a DL-based system capable not only of grading severity but also of mapping the spatial distribution of inflammation across colonic segments, overcoming the limitations of global scores [78]. Real-time systems confirmed high accuracy in predicting histological remission and clinical relapse, with AI-predicted active disease associated with significantly higher relapse rates compared with AI-predicted healing (28.4% vs. 4.9%, *p* < 0.001) [79,80]. In real-world settings, the EndoBRAIN-UC system demonstrated high sensitivity and specificity in predicting histological healing [81].

Beyond categorical indices, newer AI-derived metrics aim to improve sensitivity to therapeutic change. The PICaSSO histologic remission index (PHRI) demonstrated strong correlation with established histologic and endoscopic indices and excellent inter-rater agreement (ICC = 0.84) [82]. The red density (RD) score provided an operator-independent computer vision approach correlating with MES, UCEIS, and RHI [83]. Continuous grading systems such as the UC endoscopic gradation scale (UCEGS) and the cumulative disease score (CDS) offer refined quantification of inflammatory burden and improved discrimination of treatment response in clinical trials [84,85]. More recently, the Ulcerative Colitis Severity Classification and Localized Extent (UC-SCALE) enabled automated spatial assessment of severity and disease extent with strong agreement with central reading and correlation with biomarkers [86].

In CD, AI integration has been particularly impactful in capsule endoscopy (CE). CNN-based systems have demonstrated sensitivity exceeding 90% in detecting ulcers, strictures, and bleeding while significantly reducing reading times [87,88,89]. Real-world validation confirmed improved efficiency without compromising diagnostic sensitivity [90]. A recent meta-analysis of 11 AI models applied to CE reported pooled sensitivity and specificity of 94% and 97%, respectively [91]. AI has also been integrated into pan-enteric capsule platforms such as the PillCam™ Crohn’s system, achieving high sensitivity (90%) and specificity (96%) for ulcer and erosion detection across the small bowel and colon [92]. Automated calculation of validated capsule scores, including the LS and CECDAI, further enhances objectivity and workflow efficiency [93].

For ileocolonoscopy in CD, automated quantification of mucosal ulceration has shown strong correlation with SES-CD and superior predictive performance for clinical remission [94]. AI has also been applied to pCLE, where combined CNN–long short-term memory (LSTM) models achieved 95.3% accuracy in distinguishing inflamed from healed mucosa [95]. The recently developed Endoscopic ulcer Activity Score for Evaluating Crohn’s disease (EASE-CD), focused on quantifiable ulcer burden, represents a step toward AI-compatible, quantitative scoring systems [96].

From a clinical standpoint, AI has the potential to reduce variability, support real-time therapeutic decisions, decrease central reading burden in trials, and standardize severity assessment in daily practice. However, most systems require further multicenter prospective validation and integration into routine workflows before widespread adoption.

Table 1 and Table 2 summarize the principal studies investigating the application of artificial intelligence in ulcerative colitis and Crohn’s disease.

### 5.2. AI for Dysplasia Detection and Characterization

In IBD surveillance, AI aims to improve detection and characterization of subtle, flat neoplastic lesions within a background of chronic inflammation. Table 3 provides a summary of the pivotal studies investigating the performance of AI systems for dysplasia detection and characterization.

Early case reports demonstrated that systems such as EndoBRAIN could identify small dysplastic lesions subsequently confirmed histologically [114,115]. A computer-aided detection (CADe) model trained specifically on IBD-associated lesions achieved high sensitivity (>95%) on HD-WLE images, although performance was lower on DCE images [109]. Nevertheless, real-world implementation has produced mixed results; a retrospective study reported no significant improvement in adenoma detection rate (ADR) following CADe implementation in IBD surveillance (ADR 5.2% vs. 3.8%, *p* = 0.315) [110].

Beyond detection, AI-based computer-aided diagnosis (CADx) systems have shown promising performance in real-time lesion characterization. Abdelrahim et al. developed a deep learning model capable of simultaneous detection and classification of neoplastic lesions [111]. AI-assisted histologic assessment has also been explored; a CNN model predicting p53 expression from hematoxylin–eosin slides demonstrated diagnostic accuracy comparable to immunohistochemistry [112]. In a pilot study of 97 IBD lesions, the CAD-EYE system achieved 94.8% real-time accuracy in distinguishing neoplastic from non-neoplastic lesions, with high specificity (97.6%) [113].

Although these results are encouraging, active inflammation, mucosal scarring, and pseudopolyps introduce visual complexity that may limit algorithm generalizability. Robust prospective multicenter studies are still required to clarify the true clinical impact of AI on dysplasia detection rates and long-term outcomes.

While AI is redefining objective quantification of inflammatory activity and lesion detection, optimized surveillance strategies remain essential to reduce long-term cancer risk in IBD.

## 6. Endoscopic Surveillance in IBD: Practical Integration of Detection and Characterization

**Figure 2:**
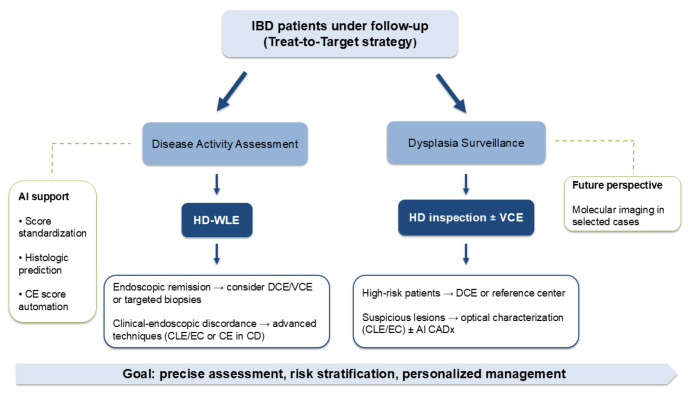
Integrated endoscopic algorithm for disease activity evaluation and dysplasia surveillance in inflammatory bowel disease (IBD) within a treat-to-target strategy. **Abbreviations: AI**, artificial intelligence; **CADx**, computer-aided diagnosis; **CD**, Crohn’s disease; **CE**, capsule endoscopy; **CLE**, confocal laser endomicroscopy; **DCE**, dye-based chromoendoscopy; **EC**, endocytoscopy; **HD**, high-definition; **HD-WLE**, high-definition white-light endoscopy; **IBD**, inflammatory bowel disease; **VCE**, virtual chromoendoscopy.

Chronic colonic inflammation in IBD is associated with an increased risk of colorectal cancer (CRC), with incidence rates approximately 1.4–1.7 times higher than in the general population [116]. The cumulative risk increases with disease duration, reaching approximately 4.5% after 20 years [117]. Although advances in disease control and surveillance have reduced dysplasia incidence, IBD-associated CRC remains a significant cause of mortality and is associated with worse survival compared with sporadic CRC (HR 1.33) [118,119]. Therefore, high-quality colonoscopic surveillance remains central to long-term management [115,120]. The integrated algorithm for disease activity assessment and dysplasia surveillance within a treat-to-target strategy is illustrated in Figure 2.

High-definition endoscopy serves as the foundation for inflammatory assessment, with escalation to enhanced imaging, targeted biopsies, or ultra-high-magnification techniques when deeper evaluation is warranted. In surveillance, careful mucosal inspection guides lesion detection, while advanced optical characterization and artificial intelligence–assisted systems may support real-time decision-making in selected cases. Molecular imaging represents a potential future extension toward biologically informed assessment. The overall aim is to enhance diagnostic accuracy, optimize risk stratification, and support personalized management.

### 6.1. Evolution from Random to Targeted Surveillance

Surveillance strategies have evolved from random four-quadrant biopsies performed at fixed intervals to targeted inspection. Random biopsies were historically justified by the limited resolution of standard-definition white-light endoscopy (SD-WLE) [7], but this approach is labor-intensive and characterized by low diagnostic yield. The introduction of HD-WLE significantly improved mucosal visualization, nearly doubling dysplasia detection compared with standard-definition systems [121].

The SCENIC consensus recommended HD-WLE or DCE over SD-WLE and suggested HD-DCE as a preferred modality where available [122]. Randomized controlled trials supported the superiority of HD-DCE in certain settings, with Alexandersson et al. reporting higher dysplasia detection rates compared with HD-WLE (*p* = 0.032) [123], and similar findings reported by Wan et al. [124]. Observational studies further supported improved detection with DCE [125,126,127].

However, other randomized and real-world studies reported no significant difference between HD-DCE and HD-WLE (*p* = 0.749 and *p* = 0.39) [128,129]. A multicenter non-inferiority trial demonstrated comparable neoplasia detection among HD-WLE, HD-VCE (i-Scan), and HD-DCE (*p* = 0.91) [130], and the VIRTUOSO trial showed no significant advantage of HD-VCE over HD-WLE (*p* = 0.14) [131]. The HELIOS trial further demonstrated non-inferiority of HD-WLE with segmental re-inspection compared with HD-DCE, suggesting that careful inspection time may be a key determinant of detection efficacy [132]. Nonetheless, a recent network meta-analysis of 26 randomized controlled trials (RCTs) identified HD-DCE as the only modality with statistically significant superiority over HD-WLE for detection of any dysplasia [133].

From a practical standpoint, meticulous high-definition inspection represents the minimum standard for IBD surveillance. Dye-based chromoendoscopy may be particularly valuable in high-risk patients or in centers with specific expertise, whereas HD-WLE or VCE may be sufficient in routine practice when careful mucosal inspection is ensured [7,117,134].

### 6.2. Real-Time Optical Characterization

Once a suspicious lesion is detected, accurate real-time characterization guides therapeutic decisions. Traditional morphologic classifications such as the Kudo pit pattern remain widely used, with neoplastic patterns (IIIS–V) associated with dysplasia under magnification and enhanced imaging [135]. High-definition chromoendoscopy and NBI have improved interobserver agreement and diagnostic accuracy [136]. The FACILE classification integrates morphology, vascular patterns, and inflammatory context across HD-WLE, DCE, and VCE, demonstrating high sensitivity (94%) for neoplasia, though moderate specificity [137].

CLE, discussed in Section 4 for inflammatory assessment, also extends its utility to surveillance. In a landmark randomized trial, chromoendoscopy combined with CLE detected nearly fivefold more neoplastic lesions than conventional colonoscopy (*p* = 0.005) while reducing biopsy numbers by 50% [138]. Probe-based CLE combined with NBI achieved 100% sensitivity and 83% specificity in distinguishing UC-associated neoplasia from regenerative lesions [139]. A meta-analysis confirmed CLE as the most accurate modality for real-time lesion characterization in IBD (sensitivity 91%, specificity 97%, AUROC 0.98) [140]. However, cost and technical demands limit widespread routine use.

EC provides ultra-high-magnification visualization of nuclear features. Integration of pit pattern analysis with endocytoscopic nuclear assessment significantly improved specificity (58% to 84%) and overall accuracy (67% to 88%) compared with pit pattern alone, particularly in patients with quiescent inflammation [141,142]. Although promising, EC requires further multicenter validation before standardized integration into surveillance algorithms.

Overall, surveillance in IBD has transitioned toward targeted high-definition inspection complemented by real-time optical characterization. The challenge for clinical practice lies in balancing diagnostic precision, procedure time, cost, and availability while ensuring adherence to evidence-based surveillance strategies.

Despite advances in detection and characterization, surveillance remains fundamentally morphology-driven, highlighting the need for biologically informed approaches.

## 7. Molecular Imaging and Predictive Targeting in IBD

While advanced imaging and artificial intelligence aim to improve visualization and standardization, molecular endoscopy represents a conceptual shift from morphology-based assessment to biologically driven visualization. Unlike conventional high-definition endoscopy, which detects structural abnormalities, molecular imaging employs fluorescently labeled probes—such as monoclonal antibodies, peptides, or activatable substrates—that selectively bind specific molecular targets, enabling real-time visualization of pathophysiologic processes at the cellular and subcellular level [98,99].

This approach addresses two major clinical challenges in IBD: the reliable detection of dysplasia within chronically inflamed mucosa and the prediction of therapeutic response before treatment initiation.

### 7.1. Molecular Imaging for Dysplasia Detection

Surveillance in IBD is complicated by the presence of background inflammation, architectural distortion, and regenerative changes that may obscure neoplastic transformation. Activatable fluorescent probes have been developed to enhance contrast selectively in dysplastic tissue.

A prominent example is the enzymatically activatable probe gGlu-HMRG, which remains non-fluorescent until cleaved by gamma-glutamyl transpeptidase (GGT), an enzyme overexpressed in colorectal cancer cells. Upon topical application, this probe generates a rapid fluorescent signal in dysplastic areas that may appear inconspicuous under white light, significantly improving detection sensitivity in preclinical models [100]. Similarly, fluorescently labeled peptides such as the phage-derived heptapeptide VRPMPLQ have demonstrated the ability to bind dysplastic colonocytes in ulcerative colitis, allowing real-time visualization of flat neoplastic lesions during confocal laser endomicroscopy [101].

Additional strategies include probes targeting cathepsin activity or intravenous fluorescent peptides directed against c-Met, which have shown the ability to differentiate neoplastic from inflammatory tissue with high contrast [102,103]. These approaches aim to move beyond random or purely morphology-based biopsy strategies toward biologically targeted sampling.

Although most of these data derive from early-phase or pilot studies, they illustrate the potential of molecular imaging to enhance diagnostic precision in dysplasia surveillance.

### 7.2. Molecular Imaging for Prediction of Therapeutic Response

Beyond dysplasia detection, molecular endoscopy is emerging as a tool for treatment personalization. The ability to visualize drug targets in vivo allows clinicians to assess target engagement directly within inflamed mucosa, potentially predicting therapeutic efficacy.

The pioneering study by Atreya et al. demonstrated that topical application of fluorescently labeled anti-tumor necrosis factor (TNF) antibodies during colonoscopy enabled visualization of membrane-bound TNF (mTNF)-expressing cells in Crohn’s disease. Patients with high baseline mTNF expression achieved a 92% clinical response to anti-TNF therapy at 12 weeks, compared with only 15% in those with low expression, with sustained predictive value at one year [104]. This study provided proof of concept that molecular imaging could serve as a predictive biomarker of biologic response.

This strategy was subsequently extended to anti-integrin therapy. Rath et al. visualized α4β7-integrin-expressing cells using a fluorescently labeled antibody during confocal endomicroscopy, demonstrating correlation between in vivo signal intensity and response to vedolizumab [105]. Further fluorescence-based studies confirmed dose-dependent mucosal uptake of vedolizumab and visualization of drug–target engagement in inflamed tissue, supporting a pharmacodynamic rationale for therapy optimization [106].

The integration of imaging with molecular profiling was further advanced in the Endo-Omics study, which combined probe-based confocal laser endomicroscopy with gene expression analysis to predict response to biological therapies. In this cohort, in vivo computerized pCLE analysis achieved high predictive accuracy (AUROC 0.93 in UC and 0.79 in CD). Ex vivo molecular imaging showed that high baseline mucosal binding of fluorescently labeled biologics predicted response in UC (AUROC 0.83, PPV 0.89), though not in CD. Transcriptomic analysis identified gene panels, including CXCL6, CXCL13, and ACTN1, associated with anti-TNF response (AUROC >0.7), supporting a biologically stratified therapeutic approach [107].

### 7.3. Clinical Implications and Future Integration

Molecular imaging has evolved from experimental concept to early clinical application. By enabling direct visualization of molecular targets and barrier dysfunction [97], this approach complements advanced imaging and AI-based quantification, offering a potential pathway toward truly personalized endoscopic assessment.

However, widespread implementation remains limited by technical complexity, regulatory considerations, cost, and the need for standardized protocols. Most available studies involve small cohorts, and large multicenter validation trials are required before routine integration into clinical practice.

From a practical perspective, molecular endoscopy is not yet ready to replace conventional surveillance or therapeutic algorithms. Nevertheless, it represents the most advanced frontier of IBD endoscopy, shifting the paradigm from descriptive morphology to biologically guided precision medicine.

## 8. Discussion

The evolution of IBD endoscopy reflects a progressive shift from morphology-based assessment toward biologically informed precision medicine. Although HD-WLE remains indispensable, it cannot fully capture the complexity of inflammatory burden or predict long-term outcomes. The documented discordance between endoscopic and histologic remission [21,22,23,24] and the prognostic relevance of proximal small bowel involvement in Crohn’s disease [25] highlight the intrinsic limits of surface-based evaluation.

Image-enhanced endoscopy improves visualization of subtle mucosal and vascular alterations and demonstrates stronger correlation with histology compared with HD-WLE alone [33,40,47,48,49]. In clinical practice, these techniques may refine risk stratification within apparent endoscopic remission. However, enhanced macroscopic inspection does not eliminate the microscopic gap.

Ultra-high-magnification modalities such as confocal laser endomicroscopy and endocytoscopy extend assessment to the cellular level, enabling real-time evaluation of barrier integrity and inflammatory activity [50,51,52,53,54,55,56,57,62,63,64,65,66,67]. The concept of barrier healing has shown prognostic relevance in selected cohorts [53,67], suggesting that remission should not be defined solely by macroscopic appearance. Nevertheless, cost, technical complexity, and limited availability currently restrict widespread use.

Artificial intelligence addresses another major limitation of conventional endoscopy—subjectivity. AI systems have demonstrated expert-level performance in grading ulcerative colitis severity [75,76,77,78,79,80,81], high sensitivity in capsule endoscopy for Crohn’s disease [87,88,89,90,91], and increasing capacity for spatial and quantitative disease mapping [78,85,86,93,96]. While promising, most evidence derives from retrospective or controlled environments, and prospective validation is required before routine adoption.

In surveillance, the transition from random biopsies to targeted high-definition inspection has improved dysplasia detection [122,123,124,125,126,127,128,129,130,131,132,133]. The incremental benefit of dye-based chromoendoscopy over meticulous HD-WLE remains debated, with recent data emphasizing inspection quality as a key determinant [130,131,132,133]. Optical characterization techniques further enhance real-time decision-making [138,139,140,141,142,143], though they remain largely confined to expert centers.

Molecular imaging represents the most advanced step in this continuum. By visualizing enzymatic activity, oncogenic pathways, or drug–target engagement [100,101,102,103,104,105,106], it introduces a predictive dimension to endoscopy. The ability to identify responders to biologic therapy through in vivo molecular visualization [104,105,107] exemplifies the transition from descriptive imaging to treatment personalization. However, these approaches remain investigational and require robust multicenter validation.

Overall, these technologies should be viewed as complementary rather than competing tools. High-definition inspection remains the foundation; enhanced imaging improves detection; optical biopsy refines microscopic assessment; artificial intelligence standardizes interpretation; and molecular imaging introduces predictive capability. The challenge ahead lies in integrating these modalities into practical, cost-effective clinical algorithms that genuinely improve patient outcomes.

## 9. Conclusions

Endoscopic evaluation in IBD is progressively shifting from morphology-based inspection toward biologically informed, precision-oriented assessment. Technological advances now allow clinicians not only to better visualize inflammation and dysplasia, but also to approximate histologic activity, reduce interpretative variability, and explore predictive biomarkers in vivo.

Rather than replacing conventional endoscopy, these innovations expand its clinical potential. High-definition inspection remains the foundation of care, while enhanced imaging, artificial intelligence, and molecular approaches provide complementary layers of information that may refine therapeutic decisions and surveillance strategies.

The priority moving forward is not the adoption of individual technologies in isolation, but their thoughtful integration into practical and cost-effective clinical pathways. Robust prospective validation and real-world implementation studies will determine which tools ultimately translate into measurable improvements in patient outcomes.

## Figures and Tables

**Table 1:** Main studies on artificial intelligence applications in ulcerative colitis endoscopy assessment.

Author (Year)	Study Design	Sample	Study Endpoints	Results
Stidham et al. [75] (2019)	Retrospective	3082 pts; 16,514 still images	Grading endoscopic severity (MES)	AUROC: 0.966 (MES 0-1 vs. 2–3), Sens: 83.0%, Spec: 96.0%. Agreement with MES (k = 0.84) comparable to expert reviewers
Takenaka et al. [76] (2020)	Prospective	2012 pts; 40,758 still images and 6885 biopsies	ER (UCEIS) and HR (Geboes score)	Acc (ER): 90.1%, Acc (HR): 92.9%. High agreement (k = 0.859) with biopsy
Gottlieb et al. [77] (2021)	Prospective (RCT data)	249 pts; 795 videos	Grading endoscopic severity (MES and UCEIS)	Excellent agreement with MES (QWK: 0.844) and UCEIS (QWK: 0.855)
Fan et al. [78] (2023)	Retrospective	332 pts; 5875 still images and 20 videos	Severity and spatial distribution of inflammatory activity	MES: Acc 86.5%, UCEIS (vascular pattern, erosions and ulcers, bleeding,): Acc 90.7%, 84.6%, 77.7%
Takenaka et al. [79] (2022)	Prospective	770 pts; full endoscopy videos and 900 biopsies	Endoscopic and histologic remission	Sens (HR): 97.9%, Spec (HR): 94.6%. Excellent agreement (ICC: 0.927 for UCEIS) with experts
Maeda et al. [80] (2022)	Prospective	135 pts	Clinical relapse	Higher relapse rate in AI-active group (28.4%) vs. AI-healing group (4.9%), *p* < 0.001
Omori et al. [81] (2024)	Retrospective	52 pts; 191 biopsies	Histologic remission (Geboes score)	Sens (HR): 74.2%, Spec (HR): 93.8%, Acc (HR): 77.5% comparable to MES
Iacucci et al. [69] (2023)	Retrospective	283 pts; 1090 videos	Endoscopic remission (UCEIS and PICaSSO)	AUROC 0.85 (WLE), AUROC: 0.94 (VCE), HR prediction: Acc 80–85%
Bossuyt et al. [83] (2020)	Prospective	29 pts	Endoscopic and histologic activity	Strong correlation with RHI (r = 0.74), MES (r = 0.76), UCEIS (r = 0.74)
Takabayashi et al. [84] (2024)	Retrospective	812 pts; 13,826 pairs of still images	Endoscopic severity (UCEGS)	Spearman’s correlation coefficient (MES: 0.89, experts >0.95)
Stidham et al. [85] (2024)	Retrospective (RCT data)	1096 pts	Endoscopic severity (CDS)	CDS vs. MES (hedges’ g 0.743 vs. 0.460) in detecting treatment response
Gutierrez-Becker et al. [86] (2025)	Retrospective (RCT data)	1953 pts; 4326 videos	Endoscopic severity and disease extent (UC-SCALE)	Strong agreement with central reading (*κ* = 0.80), significant correlation (*p* < 0.0001) with biomarkers

**Abbreviations:** **Acc**, accuracy; **AI**, artificial intelligence; **AUROC**, area under the receiver operating curve; **CDS**, cumulative disease score; **ER**, endoscopic remission; **HR**, histologic remission; **ICC**, intraclass correlation coefficient; **k**, kappa coefficient; **MES**, Mayo endoscopic score; **PICaSSO**, Paddington International Virtual ChromoendoScopy Score; **pts**, patients; **QWK**, quadratic weighted kappa; **RCT**, randomized controlled trial; **RHI**, Robarts Histopathology Index; **Sens**, sensitivity; **Spec**, specificity; **UC-SCALE**, Ulcerative Colitis Severity Classification and Localized Extent; **UCEGS**, Ulcerative Colitis Endoscopic Gradation Scale; **UCEIS**, Ulcerative Colitis Endoscopic Index of Severity; **VCE**, virtual chromoendoscopy; **WLE**, white-light endoscopy.

**Table 2:** Main studies on artificial intelligence applications in Crohn’s disease endoscopy assessment.

Author (Year)	Study Design	Sample	Study Endpoints	Results
Klang et al. [87] (2021)	Retrospective	27,892 CE images	Detection of strictures	Accuracy: 93.5% (±6.7%). AUROC: 0.989 (strictures vs. normal), AUROC 0.942 (strictures vs. ulcers)
Andrade et al. [88] (2025)	Prospective	259 SBCE exams	Detection of ulcers and erosions	Sens: 90.2% (vs SoC 69.6%), Spec: 84.4% (vs SoC 99.4%). Reading time: 172s vs. SoC (*p* < 0.001)
Freitas et al. [89] (2020)	Retrospective	115 patients	Evaluation of inflammatory activity (Lewis score)	Superior agreement (k = 0.92) for moderate-to-severe activity
O’Hara et al. [90] (2023)	Retrospective	40 CE patient studies	Real-world performance vs. standard reading	Sens: 98.1% (vs 86.2%, *p* < 0.001). Reading time: 2.3 min (vs 29.7 min, *p* < 0.001)
Ferreira et al. [92] (2022)	Retrospective	8085 PillCam™ Crohn’s capsule images	Detection of ulcers and erosions	Ulcers (Sens: 83.0%, Spec: 98.0%), erosions (Sens: 91.0%, Spec 93.0%)
Cardoso et al. [93] (2024)	Retrospective	61 patients	Evaluation of inflammatory activity	Strong correlation with LS and CECDAI (Spearman’s ρ = 0.751, ρ = 0.707), *p* = 0.001
Cai et al. [94] (2025)	Retrospective	4487 CE still images	Quantification of mucosal ulceration	Correlation (r = 0.73–0.85, *p* < 0.0001), with SES-CD
Udristoiu et al. [95] (2021)	Retrospective	54 patients; 6205 pCLE images	Inflamed vs. normal mucosa	Accuracy: 95.3%, Sens: 94.6%, Spec: 92.78%, AUC: 0.98

**Abbreviations: AUC/AUROC**, area under the curve/area under the receiver operating curve; **CE**, capsule endoscopy; **CECDAI**, capsule endoscopy Crohn’s disease activity index; **k**, kappa coefficient; **LS**, Lewis score; **pCLE**, probe-based confocal laser endomicroscopy; **r**, correlation coefficient; **SBCE**, small bowel capsule endoscopy; **Sens**, sensitivity; **SES-CD**, Simple Endoscopic Score for Crohn’s Disease; **SoC**, standard of care; **Spec**, specificity.

**Table 3:** Main studies on artificial intelligence applications in IBD dysplasia surveillance.

Author (Year)	Study Design	Sample	Study Endpoints	Results
Guerrero Vinsard et al. [109] (2023)	Retrospective	1692 HD-WLE and DCE images	Lesion detection (CADe)	Higher performance on HD-WLE (Sens: 95.1%, AUC: 0.85) vs. DCE (Sens: 67.4%, AUC: 0.65), better for small lesions
Goldman et al. [110] (2025)	Retrospective	975 colonoscopies	Adenoma detection rate (pre-CADe vs. post-CADe)	No significant improvement in ADR with CADe (5.2% vs. 3.8%, *p* = 0.315)
Abdelrahim et al. [111] (2024)	Retrospective	30 pts; 478 images	Lesion detection and characterization	Detection (Sens: 93.5%, Spec: 80.6%) and characterization (Sens: 87.5%, Spec: 80.6%)
Noguchi et al. [112] (2022)	Retrospective	12 pts; 25,849 patches	Prediction of p53 expression from H&E slides	High accuracy (86–91%), Sens (73–83%), Spec (91–92%), comparable to IHC
Picardo et al. [113] (2025)	Retrospective	97 lesions	Lesion characterization (CAD-EYE)	Accuracy: 94.8%, Spec: 97.6%, Sens: 80.0%

**Abbreviations: ADR**, adenoma detection rate; **AUC**, area under the curve; **CAD-EYE**, computer-aided diagnosis system (Fujifilm); **CADe**, computer-aided detection; **DCE**, dye-based chromoendoscopy; **H&E**, hematoxylin and eosin; **HD-WLE**, high-definition white-light endoscopy; **IBD**, inflammatory bowel disease; **IHC**, immunohistochemistry; **pts**, patients; **Sens**, sensitivity; **Spec**, specificity.

## Data Availability

No new data were created or analyzed in this study. Data sharing is not applicable to this article.
